# Gender Differences in Coronary Artery Disease, Clinical Characteristics, and Angiographic Features in the Jazan Region, Saudi Arabia

**DOI:** 10.7759/cureus.30239

**Published:** 2022-10-12

**Authors:** Ahmed I Sayed

**Affiliations:** 1 Internal Medicine, Jazan University, Jazan, SAU

**Keywords:** saudi, gender, coronary angiogram, clinical characteristics, : coronary artery disease

## Abstract

Background: Cardiovascular disease is a major cause of death worldwide. In Saudi Arabia and the gulf area, coronary artery disease (CAD) is considered a serious problem with high mortality. Previous studies identified multiple risk factors, that include hypertension, diabetes, dyslipidemia, obesity, smoking, and low physical activity, which might be related to lifestyle changes over the last few decades. Limited data about gender differences in clinical and angiographic characteristics among CAD patients in the Jazan region are available. The aim of this study is to assess potential gender differences in CAD and its clinical characteristics and angiographic features in the Jazan region.

Methods: This retrospective chart review collected data between January 2020 and March 2021 from the follow-ups of 498 patients (100 females and 398 males) aged over 18 years at the Prince Mohammed bin Nasser Hospital in the Jazan region of Saudi Arabia. The data were collected after all patients admitted to the hospital were reviewed, and cardiac catheterization was performed.

Results: Of the 498 patients with CAD, 100 (20.1%) were female and 398 (79.9%) were male. The mean age of female patients (59.44 years) was significantly higher than that of male patients (55.1 years; P=0.0002). In addition, risk factors differed significantly between genders (P=0.0210). Moreover, vessels differed significantly between genders (P=0.0002). Coronary angiogram findings showed significant correlations between gender and percutaneous coronary intervention (PCI; P=0.0001) and MEDICAL (P=0.0010). Diagnosis findings showed significant gender differences in STEMI (P<0.0010) and unstable angina (P<0.0010).

Conclusions: CAD severity did not differ by gender, but coronary angiogram findings showed significant relationships between gender, PCI, and MEDICAL treatment with CAD.

## Introduction

Coronary artery disease (CAD) is well known as the leading cause of morbidity and mortality worldwide [1]. Atherosclerosis is known as the primary cause of CAD, which develops early in life. In contrast, symptomatic CAD and acute coronary disease rarely occur in people younger than 40 [2]. This condition has become a major public health concern because CAD prevalence increases annually by 1% [3]. However, age-related CAD mortality has decreased in recent years due to advancements and improvements in cardiovascular medicine technology [4-7]. Numerous studies have suggested that the incidences of CAD, metabolic syndrome, hypertension, and hyperglycemia are higher at younger ages due to high-fat diets and unhealthy lifestyles [1,8].

Hypercholesterolemia, smoking (SMOK), diabetes mellitus (DM), obesity, hyperglycemia, and low high-density lipoprotein (HDL) levels and non-modifiable risk factors (e.g., age and family history) are CAD risk factors [9,10]. In addition, nonatherosclerotic CAD risk factors include cocaine use, connective tissue diseases, high homocysteine levels, and significant hyper coagulopathy such as antiphospholipid syndrome and nephrotic syndrome [2,11,12]. Women with acute coronary disease typically have worse short- and long-term prognoses than men, reflecting different baseline characteristics or physiopathology between women and men [12]. This gender bias was first investigated and described in 1991, showing that women with CAD are less likely to undergo coronary angiography and show serious clinical changes than men [6,9,13,14].

Recent observational studies have shown that the prognosis of women and men with the chronic coronary disease remains unclear [3,5]. However, few studies have focused on differences between men and women with CAD [4,15-18], comparing the angiographic findings, clinical characteristics, in‑hospital complications, and pharmacological recommendations of men and women [1,8,13,19]. Consequently, this area requires further attention. In this study, we assessed gender differences in CAD related to changes and differences in clinical findings, medications, angiographic features, and other factors that may affect the presence, occurrence, and complications of CAD.

## Materials and methods

This retrospective chart review study collected data between January 2020 and March 2021 from patient follow-ups with a particular focus on gender differences. The study cohort consisted of 498 patients (100 females and 398 males) aged over 18 years treated at the Prince Mohammed bin Nasser Hospital in the Jazan region of Saudi Arabia, the only Ministry of Health hospital with a cath lab capability. Departmental research committee approval was obtained, and the data were collected after all patients were admitted to the hospital, reviewed, and cardiac catheterization was performed.

Catheterization films and patient records were reviewed, and treatment methods such as catheterization, open-heart surgery, or only medical treatments were identified. This retrospective chart review was performed on 498 patients with ST-elevation myocardial infarction (STEMI), acute coronary disease, or stable angina (SA).

The collected data included sex, age, CAD risk factors (DM, hypertension [HTN], SMOK status, family history [FHX], and dyslipidemia [DYS]), diagnostic criteria (STEMI and its site from electrocardiography [ECG], SA, unstable angina [UA], non-STEMI [NSTEMI], medications, ejection fraction, and coronary angiogram finding), and intervention. We divided CAD severity into three groups based on the qualitative assessment of the coronary angiogram by two expert interventional cardiologists: 0, no or mild coronary atherosclerosis; 1, moderate (40%-60%) luminal stenosis; 2, severe (>70%) luminal stenosis. In addition, we categorized the CAD severity distribution by single-vessel, two-vessel, three-vessel, or left main stem (LM) disease (as it is associated with a high risk of morbidity and mortality), considering only arteries with severe stenosis (>50% for LM and >70% for all other main vessels) [20].

We performed statistical analyses to identify gender differences using Microsoft Excel (Seattle, WA, USA) and SPSS (IBS SPSS; Chicago, IL, USA) software. We compared the mean age of female and male patients at initial presentation with Student’s t-test. A two-sample Fisher’s exact test was used to compare all other categorical variables. Cramér's V is used for the correlation for nominal variables: All results with a two-sided P<0.05 were considered statistically significant.

## Results

**Figure 1:**
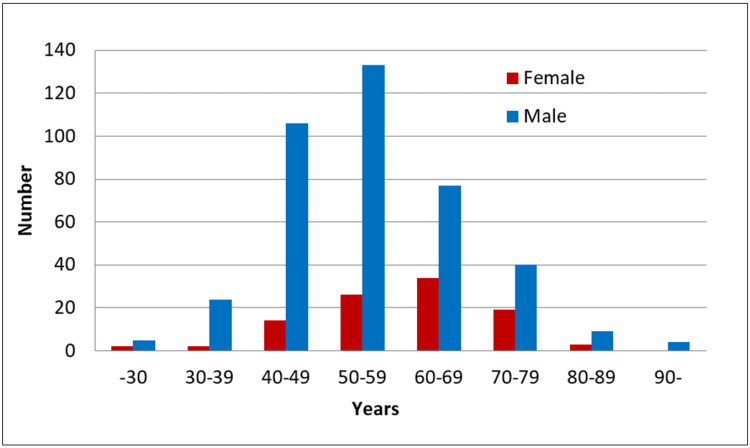
The age distribution of patients with coronary artery disease.

Of the 498 patients, who fulfilled the inclusion criteria with an initial diagnosis of CAD, 100 (20.1%) were female and 398 (79.9%) were male. The age distributions of female and male patients are shown in Figure 1, and their demographic data are presented in Table 1. The mean age at presentation was significantly higher in females (59.44 years) than in males (55.1 years; P=0.0002). In addition, females had a modal age of 60 years while men had a modal age of 50 years. The oldest male was 104 years old, and the youngest was 24, while the oldest female was 88 years old, and the youngest was 27.

**Table 1:** The demographic data (age of the groups).

Statistics Gender AGE	Female	Male	P
Total (n%)	100 (20.1%)	398 (79.9%)	0.0002
Mean	59.44	55.1	
Median	60	54	
Mode	60	50	
Std. Deviation	11.8	12.3	
Variance	138.7	150.3	
Minimum	27	24	
Maximum	88	104	

Cramér’s V indicates correlations for nominal variables, with a number between 0 and 1 indicating how strongly two categorical variables are related. The frequency of risk factors and vessels is shown in Table 2. We found that females have significantly fewer multiple risk factors than males (P=0.0210). In addition, the number of vessels was significantly lower in females than in males (P=0.0002; Table 2).

**Table 2:** Risk factors frequencies and vessel numbers. Risk factors (RF); 0: no risk factors, 1: one RF, 2: two RF, 3: three RF, 4: four RF Number of the coronary artery involved; 0: normal coronary artery, 1: one coronary artery with severe stenosis, 2: two coronary arteries with severe stenosis, 3: three coronary arteries with severe stenosis, LM: left main artery with severe stenosis

Risk factors		0	1	2	3	4	P
Female	Count	29	34	29	6	2	0.0210
	%	29.0	34.0	29.0	6.0	2.0	
Male	Count	62	131	156	34	15	
	%	15.6	32.9	39.2	8.5	3.8	
Number of vessels		0	1	2	3	LM	P
Female	Count	46	30	20	4	8	0.0002
	%	46.0	30.0	20.0	4.0	8.0	
Male	Count	103	174	75	46	25	
	%	25.9	43.7	18.8	11.6	6.3	

**Figure 2:**
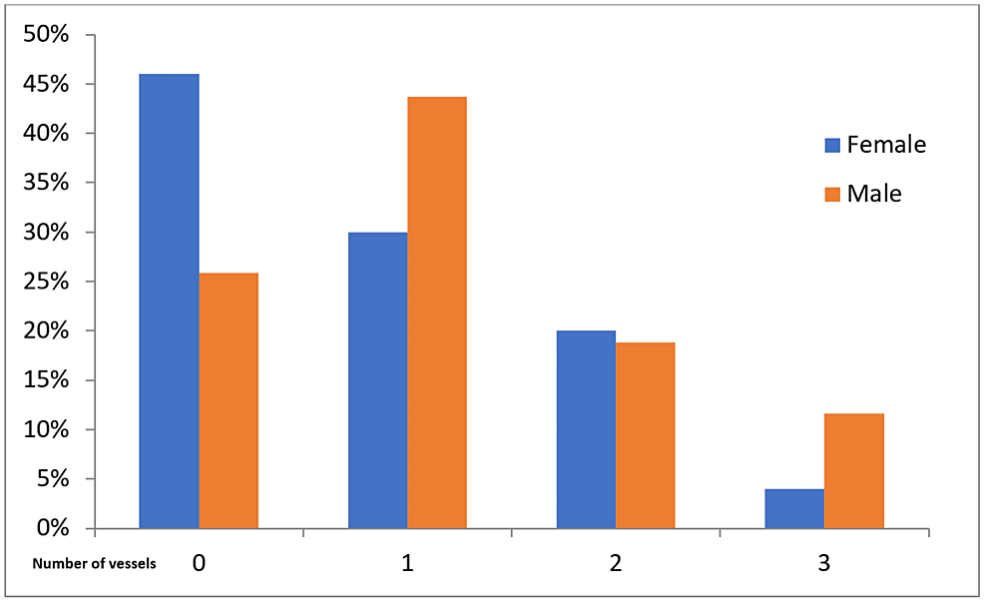
Gender disparities in vessel numbers. 0; normal, 1; one vessel disease, 2; two vessels disease, 3; three vessels disease

A visual comparison of affected vessel numbers shows that 46% of females did not need any intervention compared to 25.9% of males (Figure 2). However, 29.9% of females required percutaneous intervention in one vessel compared to 43.7% of males. Males with more affected vessels also required more percutaneous intervention (11.6%) than women (4%).

Next, the correlation coefficient Phi (ϕ) was assessed, finding that there were no significant relationships between gender and the risk factors (P>0.05; Table 3) except for SMOK (P<0.0010). However, vessel numbers differed significantly by gender with left anterior descending artery lesions (LAD; P=0.0010).

**Table 3:** Risk factors, vessels, and coronary angiogram characteristics. DM; diabetes mellitus, HTN; hypertension, SMOK; smoking, DYS; dyslipidemia, FHX; family history, STEMI; ST-elevation myocardial infarction, SA; stable angina, UA; unstable angina, NS; non-ST-elevation myocardial infarction, LM; left main, LAD: left anterior descending artery, LCX: left circumflex artery, RCA; right coronary artery, PCI; percutaneous coronary intervention, CABG; coronary artery bypass graft

	Female	Male	P
	Risk Factors		
DM	36(36%)	149(37.4%)	0.79
HTN	42(42%)	164(41.2%)	0.885
SMOK	7(7%)	143(35.9%)	<0.001
DYS	12(12%)	67(16.8%)	0.237
FHX	21(21%)	82(20.6%)	0.93
	Diagnosis		
STEMI	27(27%)	212(53.3%)	<0.001
SA	7(7%)	21(5.3%)	0.504
UA	44(44%)	82(20.6%)	<0.001
NS	22(22%)	82(20.6%)	0.759
	Vessels involved		
LM	8(8%)	25(6.3%)	0.537
LAD	34(34%)	212(53.3%)	0.001
LCX	25(25%)	118(29.6%)	0.358
RCA	23(23%)	132(33.2%)	0.05
	Management approach		
PCI	44(44%)	259(65.1%)	0.0001
MEDICAL	39(39%)	90(22.6%)	0.001
CABG	10(10%)	43(10.8%)	0.816

**Figure 3:**
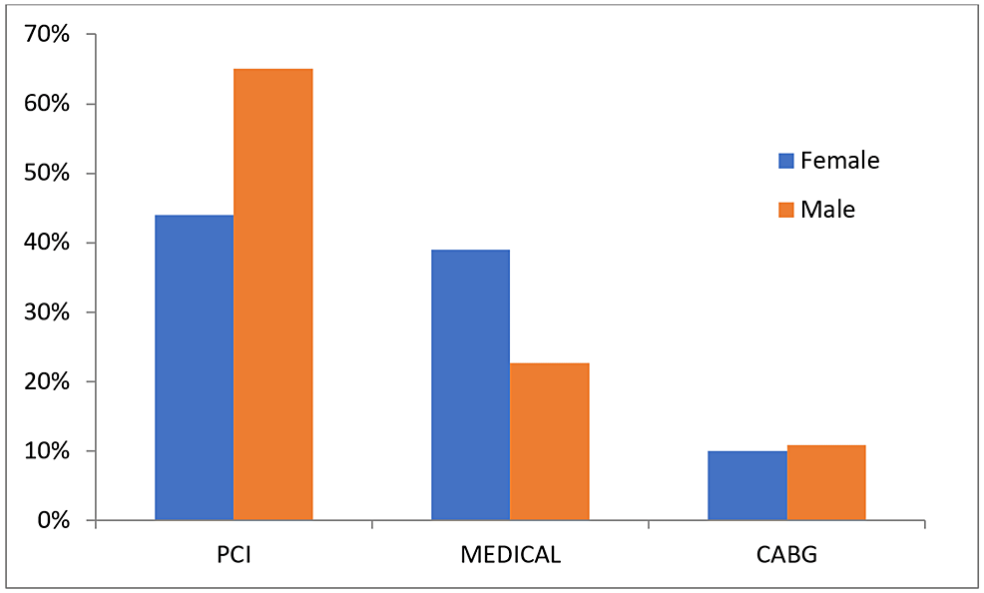
Gender disparities in Coronary angiogram findings. PCI; percutaneous coronary intervention, CABG; coronary artery bypass graft

In addition, coronary angiogram findings showed significant relationships between gender and percutaneous coronary intervention (PCI; P=0.0001) and MEDICAL (P=0.0010), with PCI higher in males (65.1%) than females (44%; Figure 3), while MEDICAL is higher in females (39%) than males (22.6%).

**Figure 4:**
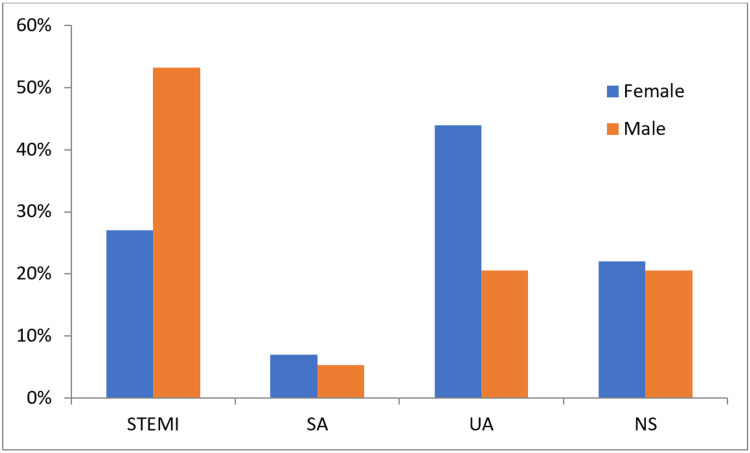
Gender differences in CAD diagnosis. STEMI; ST-elevation myocardial infarction, SA; stable angina, UA; unstable angina, NS; non-ST-elevation myocardial infarction

However, diagnosis criteria showed statistically significant gender differences and STEMI (P<0.0010) and UA (P<0.0010), with more females (44%) than males (20.6%) diagnosed with UA while more males (53.3%) than females (27%) diagnosed with STEMI in the anterior wall (Figure 4).

## Discussion

This study showed that males are more likely to present with CAD at a younger mean age (55.1 years) than females (59.44 years). However, the oldest male presented with CAD at 104 years, compared to 88 years for the oldest female. There is a linear increase in cardiovascular disease risk profile over time for males and constant evolution of the atherosclerotic process. For example, the risk of fatal CVD of only 4 % over 10 years (according to SCORE charts) in a man of 45 years of age who smokes, has an HTN, and a high cholesterol will increase to 14% when he reached 50 years of age. Conversely, the reproductive age of females can protect them from atherosclerosis since estrogen has favorable effects on the cardiovascular system [21]. However, there is an increased CAD risk in females after menopause, likely related to a combination of aging and the menopausal transition [22]. Therefore, this should be considered when performing an appropriate assessment of the CAD risk to improve the long-term CAD outcomes, guide therapy for decreasing risk, and determine the safety of menopause hormone therapy when required [22]. Moreover, Dam et al. concluded that an earlier age at menopause and surgical menopause was associated with increased CAD risk, suggesting a close monitoring requirement of such females in clinical practice [23].

Females had less frequent multiple risk factors than males in this study. However, there was no significant relationship between gender and risk factors, except SMOK status, which was higher in males. A recent review found that SMOK status was related to CAD severity and the damaged artery’s location in the heart. Nevertheless, there was no significant association between SMOK status and the number of damaged vessels and arterial occlusion location [24]. We also found significant differences between males and females in vessel numbers and the vessels involved (LAD), which was higher in males.

This study also found that females (44.0%) were more likely to be diagnosed with UA than males (20.6%), while more males were diagnosed with STEMI in the anterior wall (53.3%) than females (27.0%). A previous study concluded that females were more likely to present with atypical chest pain than males [25]. Moreover, non-invasive CAD diagnostics were less sensitive and specific in females than in males, particularly the ECG stress test [25].

Management approaches showed that the PCI rate was higher in males (65.1%) than in females (44.0%). However, medical therapy was higher in females (39.0%) than in males (22.6%). A previous study found that females had higher long-term mortality than males following PCI [26-28]. However, whether this sex survival gap was due to the older age and greater comorbidity load in females undergoing PCI or undefined sex-specific factors remained unclear [29]. Nevertheless, the cohort study of Raphael et al. on 6,847 females and 16,280 males concluded that the greater mortality following PCI in females was due to death from non-cardiac causes. This difference was accounted for by baseline age and comorbidities rather than a further sex-specific factor. [29]. However, Rao et al. concluded that there remained a difference between the outcomes of females compared with males in contemporary PCI practice, with females having significantly worse outcomes and greater mortality. There were multifactorial causes related to variances in health-seeking behavior and sub-optimal medical therapy. Females were less likely to undergo cardiac catheterization and revascularization, males were treated quicker than females, and females were less likely to receive optimal pharmacotherapy [30].

Gender differences in clinical features and outcomes of patients with various heart disorders are important in medical and social health [8]. Both females and their treating physicians are currently underappreciating their CAD vulnerability, making it difficult to provide knowledge and education and reorganize and modify practicing behaviors [1]. There are sex-specific indicators and comorbidities associated with CAD. Increased attention to cardiovascular trials, equal enrolment of females and males in large studies, and targeted preventative interventions may all assist in lowering the financial burden and negative outcomes associated with CAD in females and males with clinical and angiographic features [17]. While diagnostic, effective therapeutic, and interventional care improves the prognosis for both genders over time, clinician education is essential for ensuring that patients of both genders receive equal treatment for this extremely severe health condition [1]. Therefore, we recommend future studies to fill this gap further, resulting in better understanding and more favorable outcomes.

This study discussed a topic of global importance, which is the main cause of morbidity and mortality, while highlighting a focal area that requires more research and providing crucial insights into it. However, the generalization of its findings is limited by the cohort’s gender imbalance, retrospective nature, and single-center design.

## Conclusions

Smoking is a major risk factor particularly in males, while CAD severity is not associated with gender difference. However, the number of obstructed vessels was associated with poor prognosis and CAD severity in both genders. Coronary angiogram findings show a statistically significant relationship between males and PCI, while females are more likely to be treated medically.
